# Impact of Caloric Restriction on Molecular and Functional Networks in Rhesus Monkeys

**DOI:** 10.1093/geroni/igab046.441

**Published:** 2021-12-17

**Authors:** Rozalyn Anderson

**Affiliations:** University of Wisconsin-Madison, Madison, Wisconsin, United States

## Abstract

Caloric restriction (CR) delays aging and the onset of age-related disease in diverse species. Several diseases of aging including diabetes, cancer, and neurodegeneration, have an established metabolic component. Although the mechanisms of CR remain unknown, numerous factors implicated in longevity regulation by CR converge on regulation of metabolism. The reprograming of metabolism with CR is tissue specific, but mitochondrial activation and changes in redox metabolism are among the shared features. Changes in non-coding miRNA and in processing of transcripts are contributing mechanisms in integrating metabolic and growth pathways. Our studies in simple cell culture shows that small changes in metabolic status can precipitate large-scale multi-modal functional changes across cellular processes. We propose that modest failures in metabolic integrity with age broadly impact homeostasis and adaptation, creating shared vulnerability to diseases and conditions despite differences in their etiology, and that CR harnesses this same axis to promote health and enhanced longevity.

